# Combined Periodontal-Orthodontic Treatment with Periodontal Corticotomy Regenerative Surgery in an Adult Patient Suffering from Periodontitis and Skeletal Class II Malocclusion: A Case Report with 5-Year Longitudinal Observation

**DOI:** 10.3390/medicina60060904

**Published:** 2024-05-29

**Authors:** Peihui Zou, Gang Yang, Hao Liu, Li Gao, Qingxian Luan

**Affiliations:** 1 Department of Periodontology, School and Hospital of Stomatology, Peking University, NO. 22, Zhongguancun South Avenue, Haidian District, Beijing 100081, China; zphntyc@163.com (P.Z.); yanggang@pkuss.bjmu.edu.cn (G.Y.); 2 National Center for Stomatology & National Clinical Research Center for Oral Diseases & National Engineering Research Center of Oral Biomaterials and Digital Medical Devices& Beijing Key Laboratory of Digital Stomatology & NHC Key Laboratory of Digital Stomatology & NMPA Key Laboratory for Dental Materials, Beijing 100081, China; 3 Department of Orthodontics, School and Hospital of Stomatology, Peking University, NO. 22, Zhongguancun South Avenue, Haidian District, Beijing 100081, China; liuhao5007@126.com

**Keywords:** combined periodontal-orthodontic treatment, periodontal corticotomy regenerative surgery, periodontal phenotype, skeletal Class II malocclusion, periodontitis, fenestration and dehiscence

## Abstract

A thick periodontal phenotype with thick gingiva and alveolar bone volume is required for safe orthodontic tooth movement and long-term stability. A high incidence of dehiscence and fenestration in the labial aspect of mandibular anterior teeth may limit the correction of deformity and orthodontic treatment, especially when the lower anterior teeth are needed to have a large range of movement. This study reports a combination of periodontal therapy and orthodontic therapy with periodontal corticotomy regenerative surgery (PCRS) in a 25-year-old patient suffering from skeletal Class II malocclusion and periodontitis. The patient received periodontal therapy 5 years ago and commenced orthodontic treatment 4.5 years ago. During the 4 years of follow-up for PCRS, the clinical and radiographic evaluations revealed significant improvements in the periodontal phenotype of the mandibular anterior region. The periodontal phenotypes in the mandibular incisors region were all modified from thin to thick. Supplementing orthodontic treatment with labial PCRS could be a promising treatment strategy to maintain long-term periodontal health in adult patients with alveolar deficiency and thin gingiva tissue.

## 1. Introduction

Skeletal Class II malocclusion is a common type of malocclusion manifested as a protrusive maxilla and/or a retrusive mandible with a convex profile and distal molar relationship [1]. Some cases may present with a deep overbite, deep overjet, and gummy smile [1,2]. The success of orthodontic treatment depends on whether the tooth can be moved to the desired position, and the mandibular incisors often need to be moved labially in patients with skeletal Class II malocclusion [2,3]. However, the anatomical limitations of the alveolar bone housing pose a challenge to successful orthodontic tooth movement. The insufficient inadequate alveolar bone volume and thickness cannot support a large range of tooth movement [4,5]. Tooth movement exceeding the confines of the alveolar bone envelope may cause contact between the tooth root and cortical bone, leading to bone fenestration, bone dehiscence, gingival recession, root resorption, and black triangle [3,6].

Periodontitis commonly presents with alveolar bone reportion and compromised periodontal supportive tissue. The condition usually occurs in adults and affects oral function and aesthetics [7]. Moderate to severe periodontitis incurs an elevated risk of pathologic tooth migration and malocclusion, which may require multidisciplinary treatment, including difficult orthodontic treatment [7,8]. With the rising demand for adult orthodontics, achieving safe orthodontic tooth movement in patients suffering from periodontitis and malocclusion represents a worldwide issue that needs to be solved urgently.

Periodontal corticotomy regenerative surgery (PCRS), also known as periodontally accelerated osteogenic orthodontics (PAOO), has been used extensively to accelerate orthodontic tooth movement by providing adequate alveolar bone support, especially in Class III malocclusion [2,5,9,10,11,12]. However, only a limited number of studies have evaluated the application and effectiveness of PRCS in adult extraction cases with skeletal Class II discrepancy. A few limited long-term studies of combined periodontal-orthodontic treatment with PRCS have been documented in scholarly sources [2,10]. This case report details a patient who underwent 5 years of longitudinal observation and 4 years of follow-up for PCRS, illustrating that corticotomy on the labial side can be performed to assist orthodontics and improve the periodontal phenotype in patients with Class II malocclusion.

## 2. Case Description

### 2.1. Chief Complaints

In January 2019, a 25-year-old woman attended the Department of Periodontology of the Peking University School and Hospital of Stomatology. Her chief complaints were gingival bleeding for 2 years during tooth brushing and crowded anterior teeth in the mandibular region.

### 2.2. History of Present Illness

The patient underwent one supra-gingival scaling and the symptoms of gingival bleeding were improved. She brushed her teeth two times for 2 min each every day. She denied the use of dental floss or interdental brush. The patient was a non-smoker.

### 2.3. History of Past Illness

In addition to the above, any other dental treatment was denied by the patient.

### 2.4. Personal and Family History

A relevant family or personal history was denied by the patient.

### 2.5. Clinical Examination

**Figure 1:**
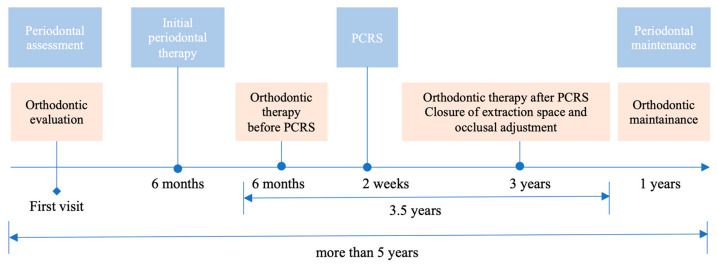
Schematic overview of the combined periodontal-orthodontic treatment used in this case. PCRS: periodontal corticotomy regenerative surgery.

**Figure 2:**
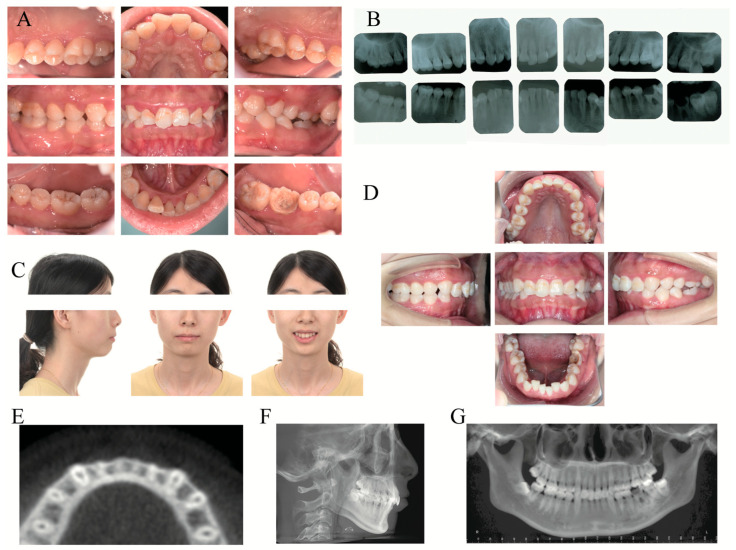
Clinical photographs and radiographs before periodontal treatment and orthodontic treatment. (**A**) Intraoral photographs before periodontal treatment. (**B**) Full-mouth periapical films. (**C**) Extraoral photographs before orthodontic treatment. (**D**) Intraoral photographs before orthodontic treatment. (**E**) Horizontal plane in cone-beam computed tomography (CBCT) at the level of 8 mm apical to CEJ of #21. (**F**) Lateral cephalogram. (**G**) Panoramic radiograph.

Extraoral examination showed a gummy smile with facial symmetry. Intraoral examination indicated poor oral hygiene with a significant amount of supragingival plaque and little subgingival calculus. Periodontal examination revealed a bleeding on probing (BOP) rate of 100% with a bleeding index of 4 (Figure 1). Most sites with a probing depth (PD) exceeding 4 mm were found at the interproximal aspect. The PD of #36 was 4 to 6 mm, exhibiting mobility of grade II with large caries and furcation involvement of grade II. Assessment of the soft tissues revealed a thin periodontal biotype with adequate keratinized gingiva width (KGW, > 2 mm) in the mandibular anterior teeth from #34 to #44 (Figure 2A–C). The orthodontic examination revealed a molar Class II relationship with mild dental crowding (the maxilla and mandible arch) as well as overbite (from #12 to #22) and buccal crossbite (#27 to #37) (Figure 2D). No pain, clicking, or other positive signs were detected in the temporomandibular joint during jaw movements.

### 2.6. Laboratory Examinations

The results of all blood tests, including complete blood count and blood clotting, and biochemical examinations such as liver function, blood glucose, and blood lipids were normal.

### 2.7. Imaging Examinations

Full-mouth periapical films showed extensive, mild, horizontal resorption of the alveolar bone, except #36 (Figure 2B). The caries of #36 were large and affected the pulp cavity, with severe bone resorption below root furcation and periapical low-density shadow (Figure 2B). Cone beam computed tomography (CBCT) demonstrated different levels of bone fenestrations in the mandibular anterior teeth (Figure 2E). The cephalometric summary (Figure 2F) and panoramic radiograph (Figure 2G) indicated the existence of maxillary protrusion and mandibular retrusion, skeletal Class II malocclusion (ANB angle, 7.1°), proclination of the upper incisors (U1-NA angle, 4.5°), lingual inclination of the lower incisors (L1-NB angle, 24.3°), and soft tissue imbalance (upper lip to E-plane, 3.1 mm).

### 2.8. Final Diagnosis and Treatment Plan

In light of the examination results, the patient was diagnosed with periodontitis (Stage II, Grade B), combined periodontic-endodontic lesions of #36, and skeletal Class II malocclusion. The periodontal treatment plan included oral hygiene instruction, professional mechanical plaque removal (PMPR) and subgingival instrumentation, tooth extraction of #36 with subsequent selective implant prosthesis, PCRS before orthodontic treatment and the movement of mandibular anterior teeth, and supportive periodontal care (SPC) [13,14]. Following the orthodontic consultation, the patient was proposed two plans: plan A and plan B. Plan A involved a combined orthodontic-orthognathic treatment, which the patient refused. The patient opted for Plan B, which comprised labial fixed-appliance orthodontics, uprighting #27 and #37, extraction of #14, #24, and #36, and mesial movement of #37 and #38 to close the extraction spaces of #36. And the patient received plan B at last.

## 3. Treatment Process

### 3.1. Initial Periodontal Treatment

**Figure 3:**
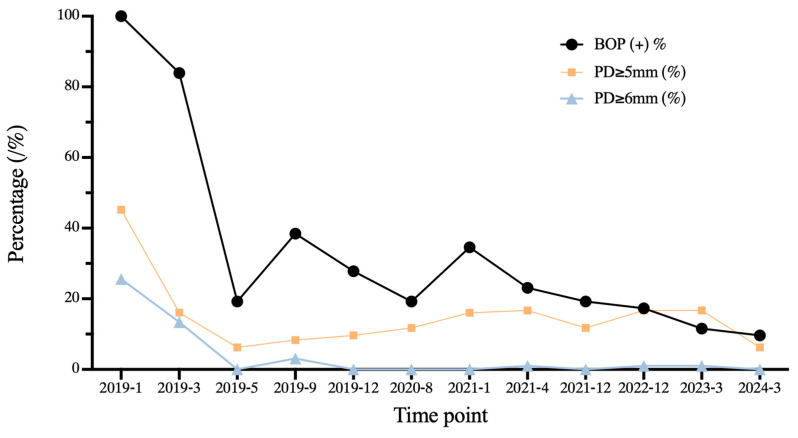
Longitudinal changes of clinical periodontal indexes according to the periodontal probing examination over 5 years of periodontal treatment. including the percentage of sites with bleeding on probing (BOP%), probing depth (PD) ≥ 5 mm, and PD ≥ 6 mm.

According to the treatment guidelines for stage I–III periodontitis [14], PMPR in step 1 and subgingival instrumentation in step 2 with ultrasound instruments and Gracey curettage were performed. Over 6 months, two rounds of periodontal re-evaluation were conducted, and #36 was extracted. The full-mouth plaque index was decreased from 80.35% to 10.71%, and the percentage of BOP-positive sites was reduced from 100.00% to 19.23%. Sites with PD = 5 mm were decreased from 19.64% to 6.25%, and sites with PD ≥ 6 mm were eliminated (Figure 3). After effectively controlling periodontal inflammation, the patient underwent orthodontic treatment.

### 3.2. Orthodontic Treatment before PCRS

**Figure 4:**
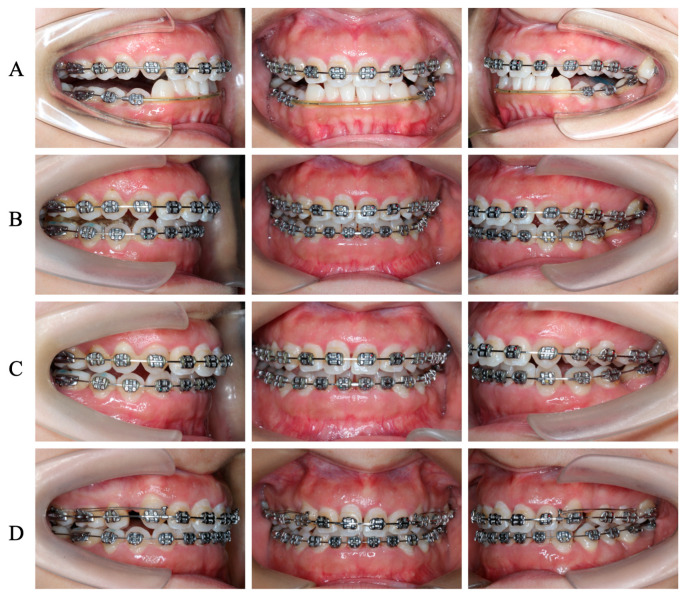
Clinical images of the detailed orthodontic process. (**A**) Initial space closure and alignment before PCRS. (**B**) Alignment of the total dental arch after PCRS. (**C**) Intrusions of #27 and #37. (**D**) Extraction space closures of #14 and #24 with finely occlusal adjustment.

Straight-archwire appliances (SHINYE, Hangzhou, China) were placed on both arches, except the region of mandibular anterior teeth (Figure 4A). The archwire sequences used for initial alignment and leveling were 0.012′′ NiTi, 0.016′′ NiTi, 0.016 × 0.022′′ NiTi, 0.018 × 0.025′′ NiTi, and 0.019 × 0.025′′ NiTi, respectively (Figure 4A–D).

### 3.3. PCRS with Piezoelectric Devices

**Figure 5:**
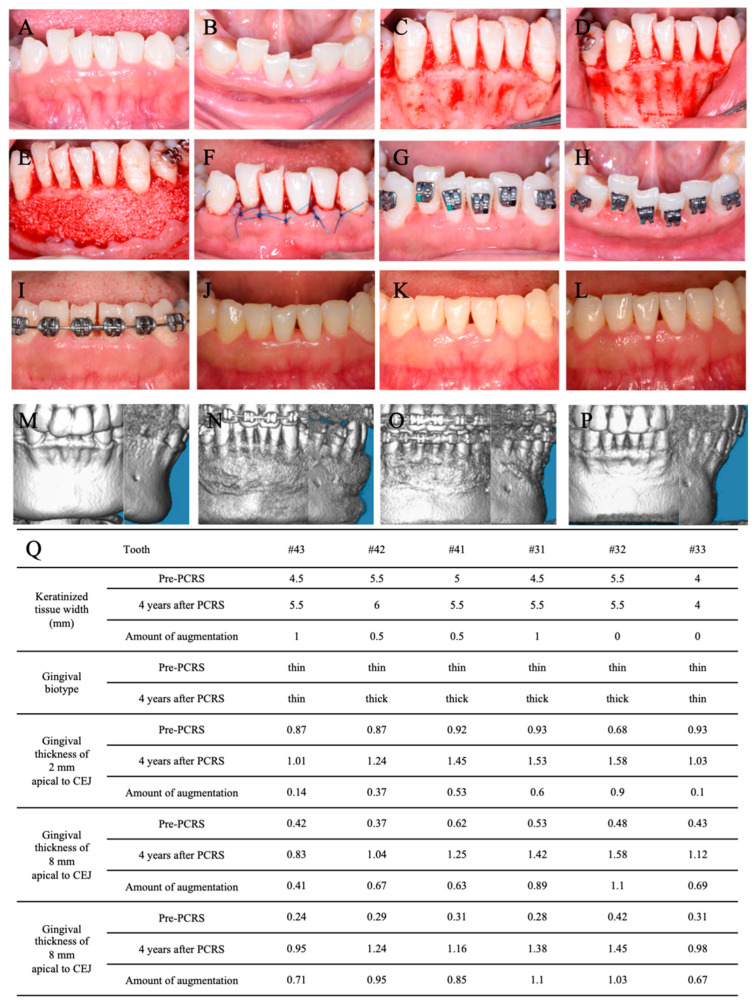
Clinical photographs of PCRS and contrast of pre-PCRS and post-PCRS in the mandibular anterior region. (**A**) Anterior view before PCRS. (**B**) Occlusal view before PCRS. (**C**) The gingival flap was elevated after papillary preservation incisions. (**D**) Piezoelectric corticotomy. (**E**) The bone graft was placed on the bone surface. (**F**) Sutures. Sutures were removed at 2 weeks after PCRS from the anterior view (**G**) and occlusal view (**H**). Six months (**I**), 2 years (**J**), 3 years (**K**), and 4 years (**L**) after PCRS. Three-dimensional reconstruction by CBCT before PCRS (**M**), immediately (**N**), 6 months (**O**), and 4 years (**P**) after PCRS. (**Q**) The clinical measurement and contrast of keratinized gingiva width and gingival biotype with gingival thickness before PCRS and 4 years after PCRS.

A labial full-thickness flap was elevated under local anesthesia with 3.4 mL articaine HCl with 1:100,000 epinephrine (Primacaine; Acteon Pharma, Bordeaux, France) (Figure 5A–C). Sunken alveolar bone was observed in the apical 1/3 of the mandibular incisors and bone dehiscence 8 mm apical to CEJ was present in all (Figure 5C). Cortical perforation was conducted at the region between the roots and the level of 3 mm apical to the apex (Figure 5D) by ultrasonic osteotome (Mectron, Carasco, Italy). The 0.75 g bone xenografts (Bio-Oss, Geistlich) mixed with autologous blood were placed on the labial alveolar bone (Figure 5E). Subsequently, the flaps were closed by simple interrupted sutures (5–0, polypropylene, 3/8 reverse cutting, Ethicon, NJ, USA) (Figure 5F).

The patient was treated with amoxicillin clavulanate potassium (457 mg, 2 times per day) for 6 days and ibuprofen tablets (300 mg, as needed) for 5 days. The patient was instructed to rinse twice daily with 0.12% chlorhexidine for 3 weeks. The sutures were removed after two weeks (Figure 5G,H) and the patient was reviewed every 1 to 6 months (Figure 5I–Q). 

### 3.4. Postoperative Orthodontic Treatment and Maintenance

**Figure 6:**
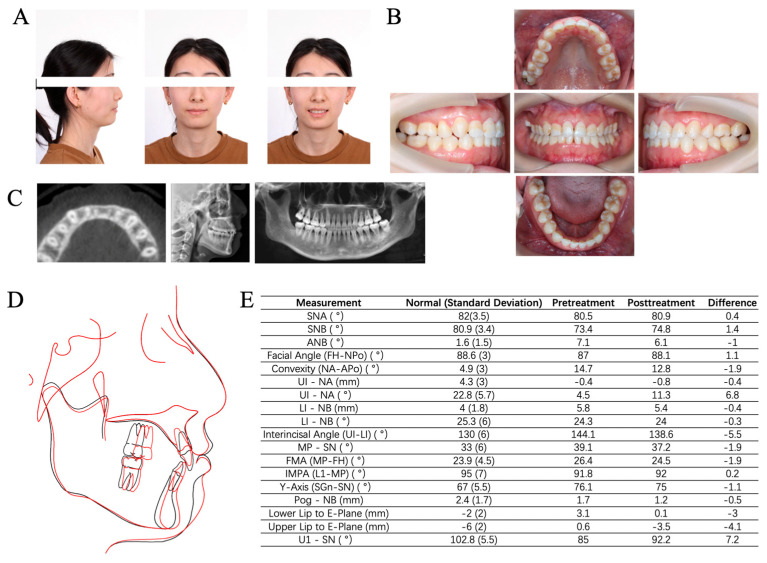
Clinical photographs and radiographs after orthodontic treatment. (**A**) Extraoral photographs. (**B**) Intraoral photographs. (**C**) Radiographs included the horizontal plane in CBCT at the level of 8 mm apical to CEJ of #21, lateral cephalogram and panoramic radiograph. (**D**) Cephmetric tracings (black: before treatment; red: after treatment). (**E**) Contrast of cephalometric measurements. SN: sella –nasion plane. FH: Frankfort horizontal plane. Po: pogonion. MP: mandibular plane. OP: occlusal plane. PP: palatal plane. Gn: gnathion.

Under local anesthesia with 1.7 mL articaine HCl with 1:100,000 epinephrine, the mini-implant (Zhongbang, Xi’an, China) was placed at the palatal side between #26 and #27 to provide anchorage for intrusion and palatal movement of #27 (Figure 4B). Rocking chair archwire (0.019 × 0.025 SS NiTi) was used to level the Spee curve. Then, 1.5 years after PCRS, #14 and #24 were extracted. Class II elastics were used to achieve the mesialization of #37 and #38, which closed the extraction space of #36 (Figure 4C). En masse space closure procedures were performed with sliding mechanics (Figure 4D). After fine occlusal adjustment and orthodontic treatment finishing, the patient was prescribed wearing thermoplastic retainers during the day and Hawley retainers at night to maintain post-orthodontic occlusion stability. A regular post-orthodontic examination was conducted once per year (Figure 6A–E).

### 3.5. Periodontal Maintenance

To achieve better plaque control and management of periodontal inflammation, SPC was performed every 4 to 9 months throughout the whole orthodontic treatment phase (Figure 3).

### 3.6. Outcome and Follow-Up

**Figure 7:**
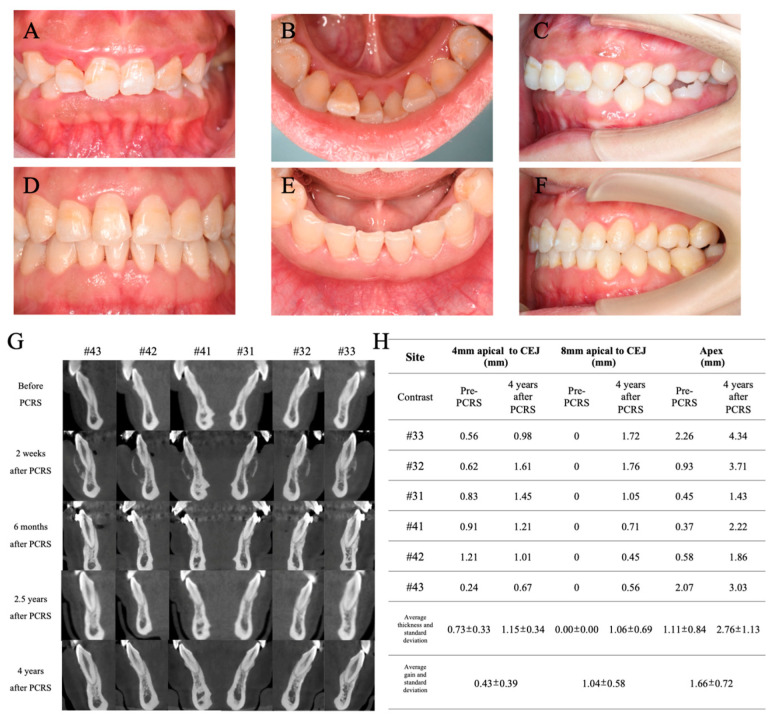
Contrast of clinical photographs and radiographs before and after the combined treatment. Frontal occlusal photograph (**A**), lingual photograph (**B**) and lateral occlusal photograph (**C**) in the first visit. Frontal occlusal photograph (**D**), lingual photograph (**E**) and lateral occlusal photograph (**F**) after 5 years of combined periodontal and orthodontic treatment. (**G**) Comparison of the sagittal plane in CBCT of the mandibular anterior region before and after PCRS. (**H**) Comparison of labial alveolar bone thickness between pre-PCRS and 4 years after PCRS at different levels.

The combined periodontal-orthodontic treatment with PCRS lasted for 4 years and the patient was reviewed for more than 1 year (Figure 1). The patient expressed satisfaction with the overall treatment outcome and reported no discomfort after PCRS surgery. The gummy smile disappeared, the anterior teeth were aligned, and the anterior overbite and posterior buccal crossbite were corrected without extra orthognathic surgery (Figure 7A–F). The extracted space of #36 was replaced by #37 without requiring implant surgery. Final cephalometric analysis (Figure 6E) revealed that SNB was increased by 1.4° and ANB was reduced by 1.0°. The decrease in MP-SN (1.9°) and MP-FH (1.9°) presented a counterclockwise rotation of the mandibular plane. A 3 mm reduction in the lower lip position relative to Ricketts’s E plane indicated a significant improvement in the lateral facial profile (Figure 6A,D,E).

After 5 years of the first visit, the full-mouth plaque index was decreased from 80.35% to 11.16%, the percentage of BOP-positive sites decreased from 100% to 9.61%, and the percentage of sites with PD = 5 mm decreased from 19.64% to 6.25%, which remained stable compared to 6 months after the initial periodontal treatment. Sites with PD ≥ 6 mm were eliminated (Figure 3), demonstrating huge improvements in oral hygiene and long-term periodontal health.

As shown in Figure 7A–F, no additional gingival recession was observed in the mandibular region until 4 years after PCRS surgery. When the Michigan periodontal probe was used to probe the gingival sulcus, the probe profile was not visible in the mandibular incisors region. The average augmentation in gingiva thickness reached 0.44 mm (2 mm apical to CEJ), 0.73 mm (4 mm apical to CEJ), and 0.89 mm (8 mm apical to CEJ), as shown in Figure 5Q. CBCT imaging and measurement revealed significant increases in labial alveolar bone thickness for six mandibular anterior teeth (#33 to #43) (Figure 7G,H). At the level of 4 mm apical to CEJ and 8 mm apical to CEJ and the apex, the initial average alveolar bone thicknesses of 0.73 ± 0.33 mm, 0 ± 0 mm, and 1.11 ± 0.84 mm were increased to 1.15 ± 0.34 mm, 1.06 ± 0.69 mm, and 2.76 ± 1.13 mm, respectively. At the level of cervical 1/3, middle 1/3, and apex 1/3 of the root, the alveolar bone thickness gains reached 0.43 ± 0.39 mm, 1.04 ± 0.58 mm, and 1.66 ± 0.72 mm, respectively (Figure 7H).

## 4. Discussion

This paper reports the successful combination of periodontal and orthodontic treatment in a case of periodontitis and skeletal Class II malocclusion. Despite the patient exhibiting a challenging periodontal phenotype with extensive bone fenestration in the mandibular anterior region, the thicknesses of labial alveolar bone and gingiva were effectively increased by PCRS alone. In this case, no barrier membrane or gingival augmentation was required and periodontal phenotype modification was achieved successfully. The findings in the present case suggest that the timing of soft tissue augmentation should be delayed after hard tissue argumentation (PCRS) in patients with thin gingiva and alveolar bone.

Orthodontic treatments pose significant risks in adults, particularly in Asian populations, which show a higher prevalence of alveolar bone deficiency [5,12]. Moreover, the widespread occurrence of periodontitis among adults exacerbates the risks of inadequate gingiva and alveolar bone, thereby complicating orthodontic interventions [7,9]. Establishing healthy periodontal conditions is crucial before commencing orthodontic procedures in adults afflicted with periodontitis. Hence, detailed periodontal probing of each tooth is important [8,15]. The PD of each tooth should be controlled to the level less than 5 mm, and several sites with PD = 5 mm are acceptable. Strictly, sites with PD ≥ 6 mm should be avoided [16]. Residual periodontal inflammation may accelerate alveolar bone resorption and gingival recession during orthodontic treatment. In the current case, the initial periodontal treatment lasted 6 months, ensuring the complete elimination of sites with PD ≥ 6 mm and a reduction in the percentage of BOP-positive sites to less than 20%. SRP was performed 12 times over 5 years to achieve strict control of periodontal inflammation, contributing to safe orthodontic treatment and better aesthetic results. CBCT was analyzed before treatment and 5 years post treatment, and no obvious progressive bone loss was observed.

The periodontal phenotype means the features of alveolar bone and gingiva. These features are affected by genetic and environmental factors and are determined by alveolar bone morphotype and gingival phenotype [3,17,18]. The gingival phenotype includes gingival thickness and KGW, while alveolar bone morphology is determined by the thickness of the labial/buccal bone plate [18,19]. Numerous studies have confirmed that PCRS can increase the alveolar bone thickness and keratinized gingiva width, thus improving the periodontal phenotype [2,4,9,10,20,21]. In more recent years, scholars have investigated the impact of PCRS on gingival thickness [5,22]. Our case demonstrated a significant augmentation in gingival thickness following PCRS. The increased KGW and gingival thickness after PCRS may be attributed to the following mechanisms. Firstly, performing corticotomy with a piezoelectric knife provides abundant blood vessels, which promotes postoperative healing and increases the regenerative potential of periodontal soft tissues [10,22,23]. Secondly, a previous study found guided tissue regeneration with a coronally positioned flap was able to increase KGW [24]. Despite the position of mucogingival junction (MGJ) being moved coronally, it exhibits a tendency to return to its genetic anatomical position over time, especially in patients with the congenital thick gingival biotype, thus increasing KGW under the conditions of a stable gingival margin [25]. Lastly, the phenomenon of “crawling attachment” was observed in the surgical area after PCRS, which was attributed to the proliferation and growth of fibroblasts by mechanical stimulation and the growth of granulation tissues derived from different tissues [26,27,28]. A stable blood clot between the bone graft and the gingival flap promotes mechanization and reorganization, but more related studies are needed to explore the underlying histological mechanisms. Eventually, the blood clot transforms into mature connective tissue and then increased local gingival thickness [25].

In the mandibular incisors region (#32–#42), there was a change in the labial gingival biotype from thin to thick, showing an average increase in gingival thickness of 0.60 mm to 0.98 mm, measured by periodontal probing and mucosal puncture with a root canal K-file under local anesthesia (Supplementary Figures S1 and S2). These results were consistent with Xu’s series studies [12,29]. In the mandibular canines region (#33 and #43), although the labial gingival biotype showed no obvious changes, the average increases in gingival thickness reached 0.12 mm, 0.55 mm, and 0.69 mm at the levels of 2 mm, 4 mm, and 8 mm apical to CEJ, respectively. The increase in gingival thickness in the canine region was less than that in the incisor region. There were two possible reasons for this result. On the one hand, the gingival thickness was strongly correlated with the alveolar bone morphology, and the labial alveolar bone with the thin gingival biotype was also thinner [19]. The thinner labial alveolar bone in canines may lead to less of an increase in gingival thickness compared to that of incisors. On the other hand, the width of keratinized gingiva was correlated with gingival thickness [30]. Thick gingiva often had wider keratinized gingiva, and the smaller width of the keratinized gingiva after PCRS in canines may be related to their thinner gingiva than that of incisors.

Interestingly, at 2 mm and 4 mm apical to CEJ, the amounts of gingival thickness gain of six teeth were higher than those reported in Ye Han’s studies (0.43 ± 0.35 mm and 0.33 ± 0.25, respectively) [5]. The changes in gingival thickness were first recorded at 8 mm apical to CEJ as all six teeth demonstrated bone fenestrations and thin gingiva mucosa with distinct root shapes at this location. Sunken alveolar bone was observed in the apical 1/3 of the root during PCRS, which facilitates bone augmentation and blood clot stabilization. Stable labial bone volume at this level may promote gingival augmentation in the 4 years following PCRS.

Nonetheless, the application and necessity of barrier membranes in PCRS remain controversial. The barrier membrane has been reported to prevent connective tissue growth into the bone defect and significantly enhance new bone volume and height, especially in unfavorable bone defects [31,32]. However, previous studies have also used periosteum alone and achieved satisfactory results [10,33]. The periosteum-covering technique without the barrier membrane offers enhanced graft stabilization and effective vertical bone augmentation compared to the bioresorbable collagen membrane coverage technique in the mandibular anterior tooth region [10]. In a certain way, the periosteum is a connective tissue membrane that consists of the cellular components and plenty of microvessels, which may accelerate the formation of new bone when placed in contact with the bone graft [10,33]. In patient with a thin gingival biotype, the complication of the leakage of bone graft substitutes following PCRS is more common in the absence of the barrier membrane [10]. Although no barrier membrane was used in our case, the bone graft was maintained steadily without leakage. Furthermore, the gingival flap was fully released until there was no tension, which may be important in the primary healing and long-term maintenance of bone graft contours.

Few studies have investigated the long-term efficacy of combined periodontal-orthodontic treatment with PCRS in patients with Class II malocclusion. At present, the main confounders of bone augmentation in PCRS remain indefinite and uncertain. In the present case, the labial alveolar bone thickness was the thickest at the apex level compared to 4 mm and 8 mm apical to CEJ, as previously published. PRCS effectively increased the alveolar bone volume and ensured the labial bodily movement of mandibular anterior teeth, expanding the limits of tooth movement safely (Figure 6D and Figure 7G). CBCT measurements revealed that the height and thickness of the labial alveolar bone at different bone levels were maintained from the completion of orthodontic treatment (3 years post PRCS) to 1 year after orthodontic treatment (4 years post PRCS). These results indicate the long-term benefits of PCRS for orthodontic treatment of mandibular anterior teeth. Due to the heterogeneity of each study and case, various studies use different bone grafts, including autogenous bone, allograft, xenograft (like bovine-derived particulated bone graft), and so on [5,21]. However, the type of bone graft for PRCS that yields the best clinical outcomes remains controversial [4,5,21]. More high-quality PRCS-related studies are required in the future to guide clinical decision making. In this case, bovine-derived particulated bone mineral (Bio-Oss) alone without a collagen membrane achieved a good clinical effect with significant modification of the periodontal phenotype.

## 5. Conclusions

All in all, the success of the combined periodontal-orthodontic treatment was facilitated by several key factors. First, sequential phases of periodontal treatment were adopted in this patient suffering from periodontitis, including phases of step 1, step 2, and SPC. This sequential approach provided a reasonable treatment process and guaranteed long-term treatment stability. Second, PCRS was conducted to improve the thin periodontal biotype in the lower anterior teeth, effectively eliminating bone fenestration and dehiscence and increasing the thickness of the alveolar bone, gingiva thickness, and keratinized tissue width. Therefore, orthodontic tooth movement was safer and faster. Finally, continual oral hygiene instruction and regular periodontal checkups for SPC were ensured to control periodontal inflammation during the orthodontic treatment with archwires and brackets.

Despite the limitations of our case report, these results illustrate that PCRS alone can increase the alveolar bone thickness but also increase gingival thickness, which may reduce the needs and possibilities of gingival augmentation in the future. Nevertheless, more prospective randomized controlled trials are needed to assess the reliability and stability of increased gingival thickness by conducting PCRS alone.

## Data Availability

All data supporting the findings of this study are available from the corresponding author upon reasonable request.

## Supplementary Materials

The following supporting information can be downloaded at: https://www.mdpi.com/article/10.3390/medicina60060904/s1, The methods and illustrations of measuring labial alveolar bone thickness on CBCT images and labial gingiva thickness at different levels are listed in Supplementary Figures S1 and S2.
