# Supplementary material for: Combined Periodontal-Orthodontic Treatment with Periodontal Corticotomy Regenerative Surgery in an Adult Patient Suffering from Periodontitis and Skeletal Class II Malocclusion: A Case Report with 5-Year Longitudinal Observation
Source: Medicina (Kaunas). 2024 May 29;60(6):904. doi: 10.3390/medicina60060904 (PMC11205772; doi:10.3390/medicina60060904)
Supplement: Supplementary file 1 [file medicina-60-00904-s001.zip › medicina-3023276-supplementary.pdf]

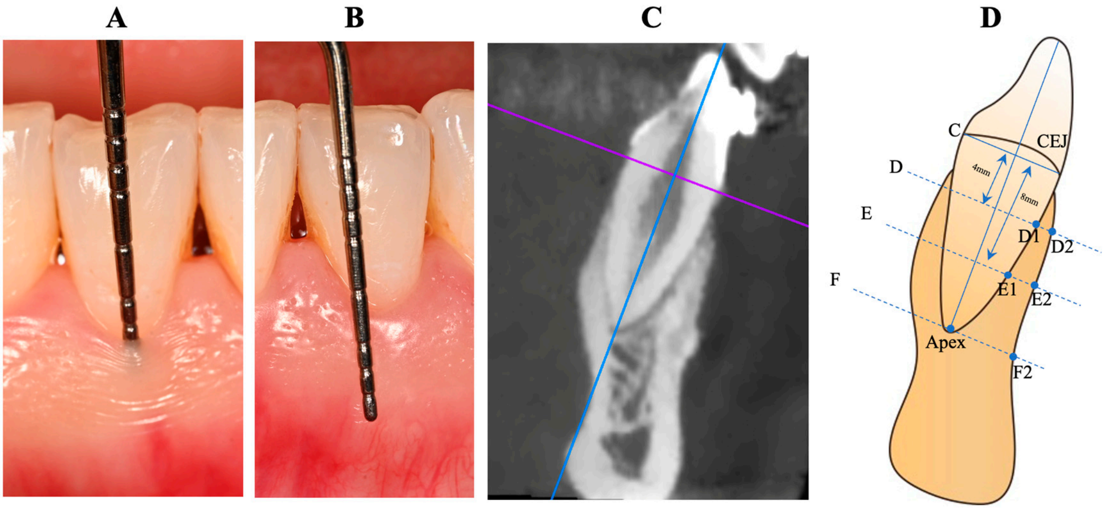

Figure S1: The example (A) and illustration (B) of measuring labial alveolar bone thickness centrally on CBCT images of the lower anterior teeth, orientated by the root long axis. Reference lines: Line D, E and F illustrate the level of 4 mm, 8 mm apical to cemento-enamel junction (CEJ) and apex level. Reference lines: Line C: the connection of points at the level of 8mm apical to cemento-enamel junction (CEJ), respectively. Reference points: D1, E1 and Apex mean the intersection of labial root surface and line D, E and F, respectively. D2, E2 and F2 mean the intersection of labial alveolar bone surface and line D, E and F, respectively. D1-D2, labial alveolar bone thickness at the level of 4 mm apical to CEJ. E1-E2, labial thickness at the level of 8 mm apical to CEJ. Apex-F2, labial alveolar bone thickness at the apex level.

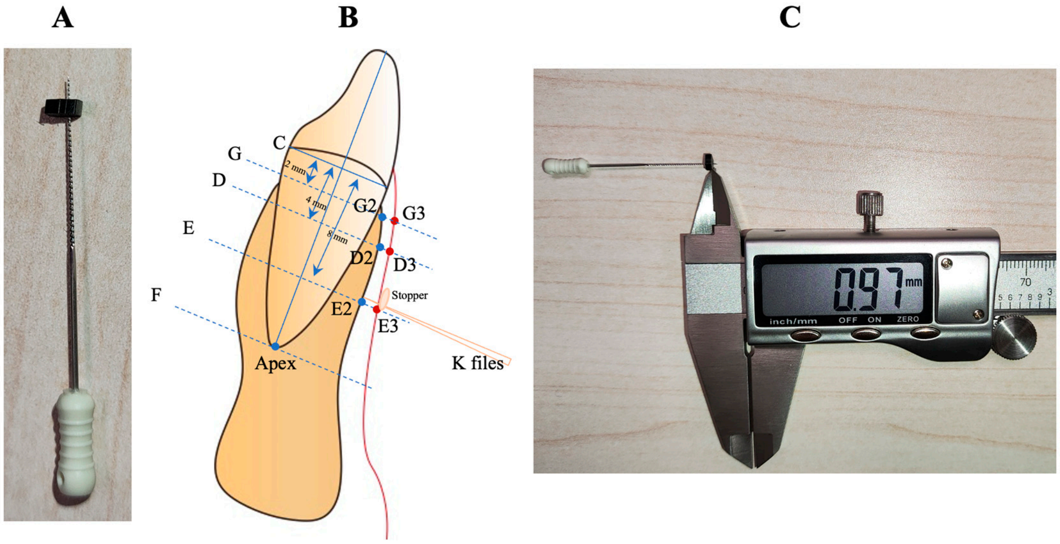

Figure S2: Under local anesthesia, the sterlized root canal K file (#15, 21 mm, MANI, Guangdong, China) were inserted in the gingival mucosa at 2 mm, 4 mm and 8 mm apical to CEJ, ensuring that the file tip contacted with the alveolar bone surface and the stopper contacted with mucosa surface (B). The distances between file tip and stopper (G2-G3, D2-D3 and E2-E3) were measured by electronic Vernier calipers (Deli, China, C).
